# Supplementary material for: Sex differences in metabolic pathways are regulated by Pfkfb3 and Pdk4 expression in rodent muscle
Source: Commun Biol. 2021 Nov 4;4:1264. doi: 10.1038/s42003-021-02790-y (PMC8569015; doi:10.1038/s42003-021-02790-y)
Supplement: Supplementary file 2 — Supplementary Information [file 42003_2021_2790_MOESM2_ESM.pdf]

Supplementary information

Sex differences in metabolic pathways are regulated by *Pfkfb3* and *Pdk4* expression in rodent muscle

Antonius Christianto[1], Takashi Baba[1,2]*, Fumiya Takahashi[1], Kai Inui[1], Miki Inoue[1,2], Mikita Suyama[3], Yusuke Ono[4,5], Yasuyuki Ohkawa[6], Ken-ichirou Morohashi[1,2]

1, Division of Molecular Life Science, Graduate School of Systems Life Science, Kyushu University, Maidashi 3-1-1, Higashi-ku, Fukuoka 812-8582, Japan
2, Department of Molecular Biology, Graduate School of Medical Sciences, Kyushu University, Maidashi 3-1-1, Higashi-ku, Fukuoka 812-8582, Japan
3, Division of Bioinformatics, Medical Institute of Bioregulation, Kyushu University, Maidashi 3-1-1, Higashi-ku, Fukuoka 812-8582, Japan.
4, Department of Muscle Development and Regeneration, Institute of Molecular Embryology and Genetics, Kumamoto University, Honjo 2-1-1, Chuo-ku, Kumamoto, 860-0811, Japan.
5, Center for Metabolic Regulation of Healthy Aging, Kumamoto University Faculty of Life Sciences, Honjo 1-1-1, Chuo-ku, Kumamoto 860-8556, Japan
6, Division of Transcriptomics, Medical Institute of Bioregulation, Kyushu University, Maidashi 3-1-1, Higashi-ku, Fukuoka 812-8582, Japan.

*Corresponding should be addressed to Takashi Baba
Department of Molecular Biology, Graduate School of Medical Sciences, Kyushu University, Maidashi 3-1-1, Higashi-ku, Fukuoka 812-8582, Japan
E-mail: takbaba@cell.med.kyushu-u.ac.jp

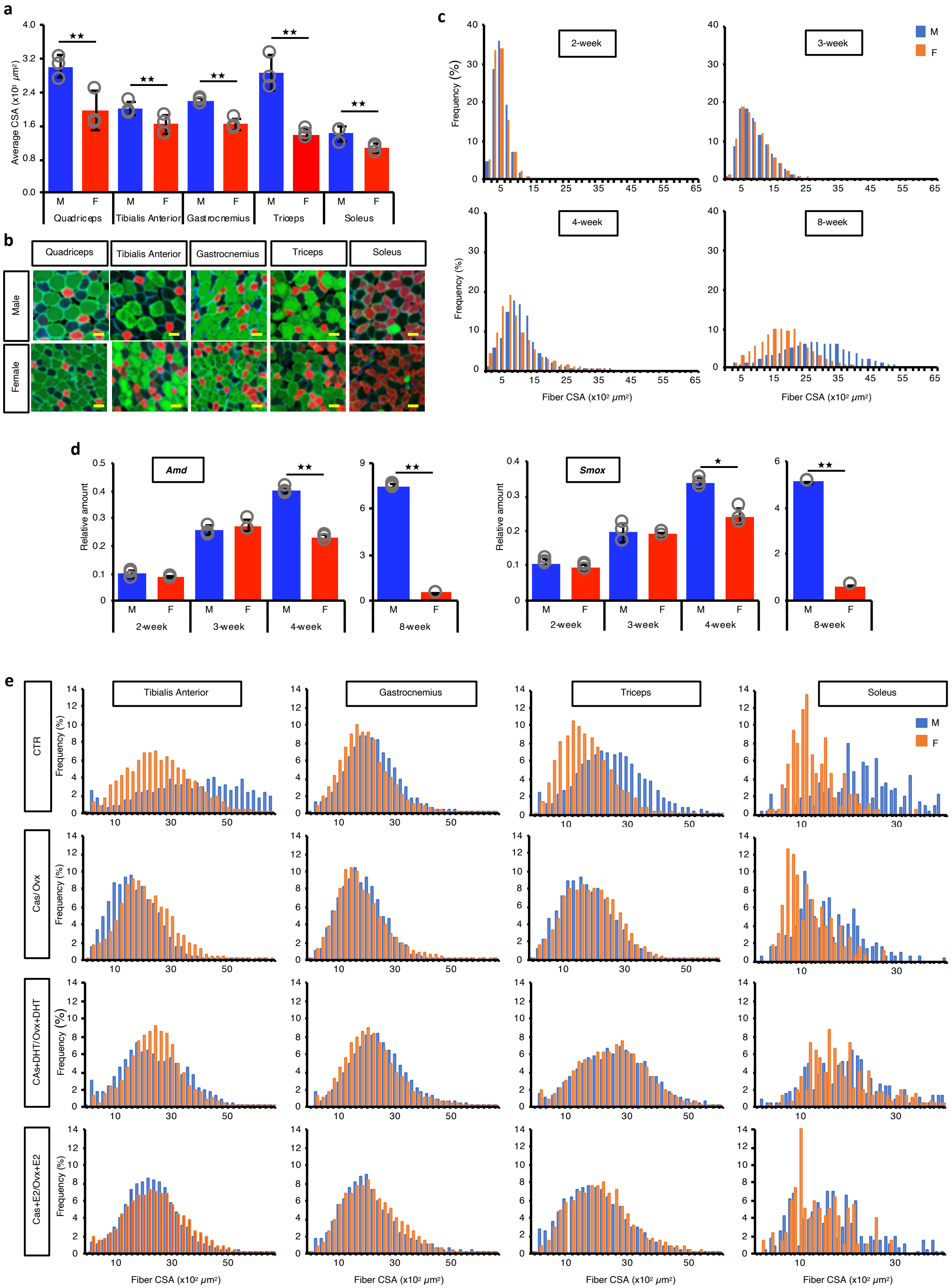
a
Average CSA (x10³ μm²)
Quadriceps
Tibialis Anterior
Gastrocnemius
Triceps
Soleus
M
F
b
Quadriceps
Tibialis Anterior
Gastrocnemius
Triceps
Soleus
Male
Female
c
2-week
3-week
4-week
8-week
Frequency (%)
Fiber CSA (x10² μm²)
d
Amd
Smox
Relative amount
2-week
3-week
4-week
8-week
e
Tibialis Anterior
Gastrocnemius
Triceps
Soleus
CTR
Cas/Ovx
CAs+DHT/Ovx+DHT
Cas+E2/Ovx+E2
Frequency (%)
Fiber CSA (x10² μm²)

Supplementary Figure 1. Sexually dimorphic features of skeletal muscles and muscle fibers.
a) Sexually dimorphic sizes of skeletal muscle fibers. Cross-sectional areas (CSAs) were determined using approximately 3000, 6000, 6000, 4000, and 50 fibers from the tibialis anterior, gastrocnemius, quadriceps, triceps, and soleus muscles of 8-week-old male (M) and female (F) mice, respectively. Three biologically independent muscle samples were used. The data are shown as means ± SD. $^{**}p<0.01$
b) Immunofluorescence staining of type IIA and IIB fibers in skeletal muscles. Type IIA (red) and type IIB (green) fibers of the skeletal muscles indicated at the figure were immunostained with antibodies against MYH2A and MYH2B, respectively. Eight-week-old male and female mice were used. Bars = 50 μm.
c) Appearance of sexually dimorphic fiber sizes in the developing quadriceps muscle. CSAs of type IIB fibers in quadriceps muscles were determined at 2, 3, 4 and 8 weeks after birth. The CSAs of more than 5000 fibers were determined for every three biologically independent samples. The distribution of the CSAs of muscle fibers from male (M) and female (F) mice are indicated. Vertical and horizontal axes indicate the frequencies (%) of type IIB muscle fibers and their CSAs, respectively.
d) Expression of Amd and Smox genes in quadriceps type IIB fibers at 2, 3, 4, and 8 weeks after birth. Total RNAs were prepared from quadriceps type IIB fibers of male (M) and female (F) mice at 2, 3, 4, and 8 weeks after birth, then subjected to qRT-PCR. Three biologically independent samples were used for qRT-PCR. The data were normalized by *Actb* and are shown as means ± SD. The expression levels of the genes at 8 weeks are higher than those at earlier time points (compare vertical units of the graphs). $^{*}p<0.05$, $^{**}p<0.01$
e) Sexually dimorphic CSAs of type IIB fibers. CSAs were determined for type IIB fibers in the tibialis anterior, gastrocnemius, triceps, and soleus muscles of sham-operated mice (CTR), gonadectomized mice (Cas, Ovx), DHT-treated mice after gonadectomy (Cas+DHT, Ovx+DHT), and E2-treated mice after gonadectomy (Cas+E2, Ovx+E2) (Fig. S2). The CSA distributions of the muscles are shown. This experiment was performed in triplicate, with essentially similar results obtained in each case. The average number of fibers analyzed in the sham-operated (CTR) males, sham-operated (CTR) females, Cas males, Ovx females, Cas+DHT males, Ovx+DHT females, Cas+E2 males, and Ovx+E2 females are presented in corresponding order for each muscle as follows: for the tibialis anterior, 2500, 2300, 3600, 3100, 4000, 3200, 3300, and 3300 fibers, respectively; for the gastrocnemius, 5900, 4600, 5700, 5400, 6200, 5100, 6700, and 4100 fibers, respectively; for the triceps, 4500, 4300, 4800, 4200, 5200, 4200, 4800, and 4300 fibers, respectively; and for the soleus, 50, 55, 65, 40, 65, 50, 63, 40 fibers, respectively. Vertical and horizontal axes indicate frequencies (%) and fiber sizes, respectively.

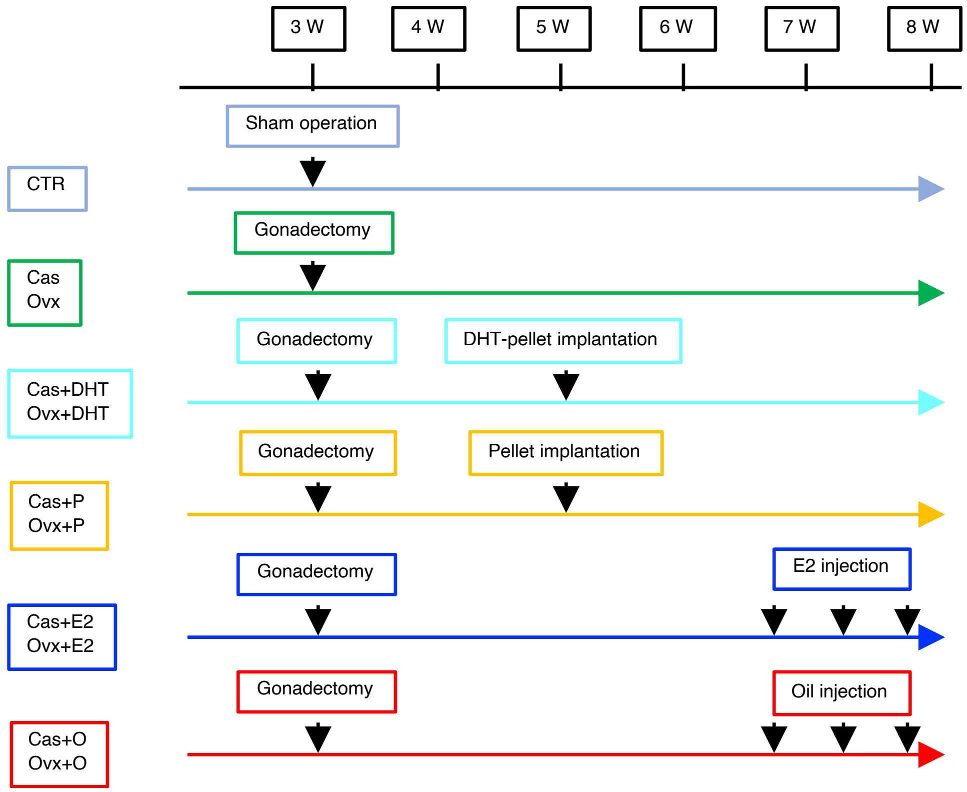

Supplementary Figure 2. Timetable for operations and sex steroid treatments.
Mice were gonadectomized (castration (Cas) and ovariectomy (Ovx)) or sham-operated (CTR) at 3 weeks after birth. A DHT pellet was implanted into gonadectomized mice (Cas+DHT and Ovx+DHT) at 5 weeks, or E2 dissolved in oil was injected three times, at 47 d, 51 d, and 55 d (Cas+E2 and Ovx+E2). As controls for the DHT and E2 treatments, an empty pellet was implanted (Cas+P and Ovx+P) and untreated oil was injected (Cas+O and Ovx+O). Skeletal muscles were prepared from these mice at 8 weeks. Skeletal muscles of eight experimental groups (male CTR, female CTR, Cas, Ovx, Cas+DHT, Ovx+DHT, Cas+E2, and OVX+E2) were used to determine CSAs, while 10 experimental groups (male CTR, female CTR, Cas+DHT, Ovx+DHT, Cas+P, Ovx+P, Cas+E2, Ovx+E2, Cas+O, and Ovx+O) were used for transcriptome studies.

a

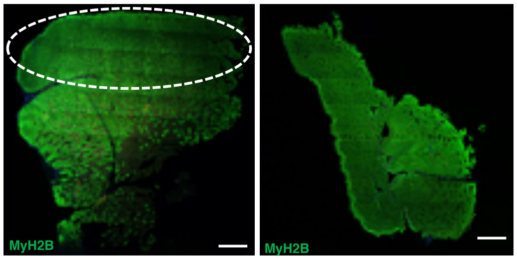

Sample		Stained	Unstained	Percentage
Male	1	4,314	89	97.9%
	2	4,770	124	97.4%
	3	5,431	80	98.5%
Female	1	4,588	189	95.9%
	2	4,991	114	97.7%
	3	5,898	387	93.4%

b

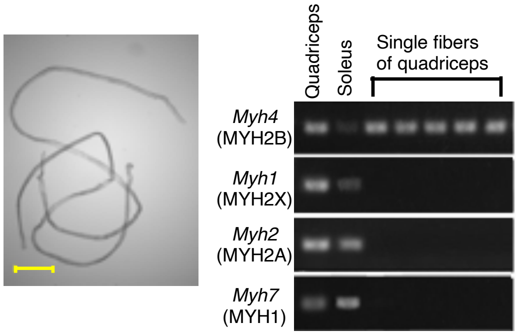

d

	μg protein/fibers Left	Right	Av	M/F
M1	18.64	18.53	18.59	
M2	19.54	19.81	19.68	
M3	19.34	19.23	19.29	1.10
F1	16.71	17.30	17.01	
F2	17.53	17.78	17.66	
F3	17.48	17.39	17.44	

c

Sample	Total reads	Uniquely mapped reads	Reads mapped to multiple loci
Female	18,025,587	14,272,388 (79.18% of total reads)	1,581,224 (8.77% of total reads)
Ovx+Oil	32,300,145	26,242,701 (81.25% of total reads)	2,231,375 (6.91% of total reads)
Ovx+P	21,370,733	17,156,100 (80.28% of total reads)	1,431,336 (6.70% of total reads)
Ovx+DHT	37,483,030	29,011,132 (77.40% of total reads)	4,054,955 (10.82% of total reads)
Ovx+E2	29,106,525	23,153,029 (79.55% of total reads)	2,034,722 (6.99% of total reads)
Male	26,602,512	20,237,863 (76.08% of total reads)	2,299,281 (8.64% of total reads)
Cas+Oil	29,637,802	23,818,569 (80.37% of total reads)	1,702,315 (5.74% of total reads)
Cas+P	22,509,281	17,795,531 (79.06% of total reads)	1,161,829 (5.16% of total reads)
Cas+DHT	45,611,883	36,634,849 (80.31% of total reads)	2,687,853 (5.89% of total reads)
Cas+E2	45,976,100	36,485,393 (79.36% of total reads)	3,039,041 (6.61% of total reads)

Supplementary Figure 3. Preparation of type IIB fibers, quality of cDNA libraries, and determination of protein amounts.

a) Preparation of the type IIB-enriched region in the quadriceps. The quadriceps muscle roughly consists of two regions with different colors, white (external region) and pale-reddish (internal region). These predominantly contain fast- and slow-type fibers, respectively. Immunofluorescence of a cross-section of the quadriceps muscle shows that most muscle fibers in the external region (enclosed by a white broken line) stain positively for MYH2B (green; left). By contrast, a substantial number of fibers in the internal region are unstained. The internal region was removed from the quadriceps muscle and a cross-section of the remaining external region underwent staining for further analysis (right). Scale bar = 500 μm. The numbers of fibers with and without MYH2B staining in the processed external region were counted in both male and female mice. Three biologically independent samples were analyzed in both sexes.

b) Determination of fiber types prepared from the quadriceps. Two individual fibers prepared from the quadriceps muscle are shown on the left. Scale bar = 200 μm. The fiber type was determined by RT-PCR using RNAs prepared from individual fibers. RNAs prepared from whole quadriceps and soleus muscles were used as controls. *Myh4* (*myosin heavy chain 4*) encoding MYH2B, *Myh1* (*myosin heavy chain 1*) encoding MYH2X, *Myh2* (*myosin heavy chain 2*) encoding

MYH2A, and *Myh7* (*myosin heavy chain 7*) encoding MYH1 were used as the marker genes for type IIB, type IIX, type IIA, and type I fibers, respectively. When whole quadriceps and soleus muscles were used, the expressions of all of these *Myh* genes were detected, although the expression level of *Myh4* was low in the soleus muscle. Only the expression of *Myh4* was detected in most of the individual fibers prepared. RT-PCR images for the five fibers are shown.

c) Evaluation of the cDNA libraries. The qualities of the cDNA libraries were evaluated based on total reads, uniquely mapped reads, and reads mapped to multiple loci.

d) Amount of protein in male and female type IIB quadriceps fibers. Muscle fibers were prepared from the left and right quadriceps of three male and three female mice. Fifty fibers were homogenized with 150 ml lysis buffer containing 150 mM NaCl, 1 mM EDTA, 50 mM Tris-HCl (pH 7.4), 2% SDS, and 10% glycerol. The amount of protein was determined using a Bicinchoninic Acid Protein Assay Kit.

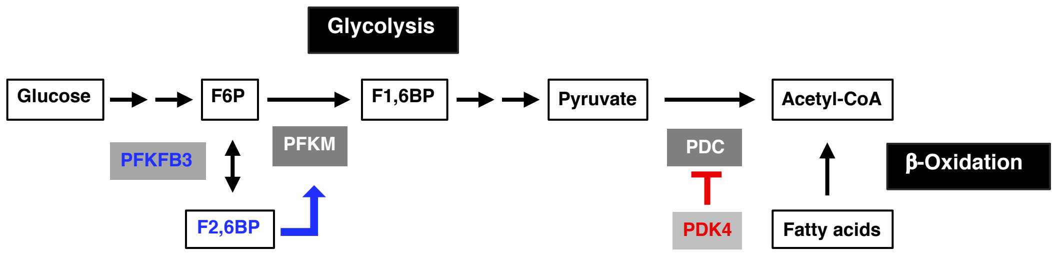

Supplementary Figure 4. The reaction mediated by PDK4.

PDK4 phosphorylates PDC and then suppresses the enzymatic activity to convert pyruvate to acetyl-CoA. Accordingly, increased activity of PDK4 results in decreased the supply of acetyl-CoA from glucose through glycolysis, and conversely increased the supply of it from fatty acids through β-oxidation.

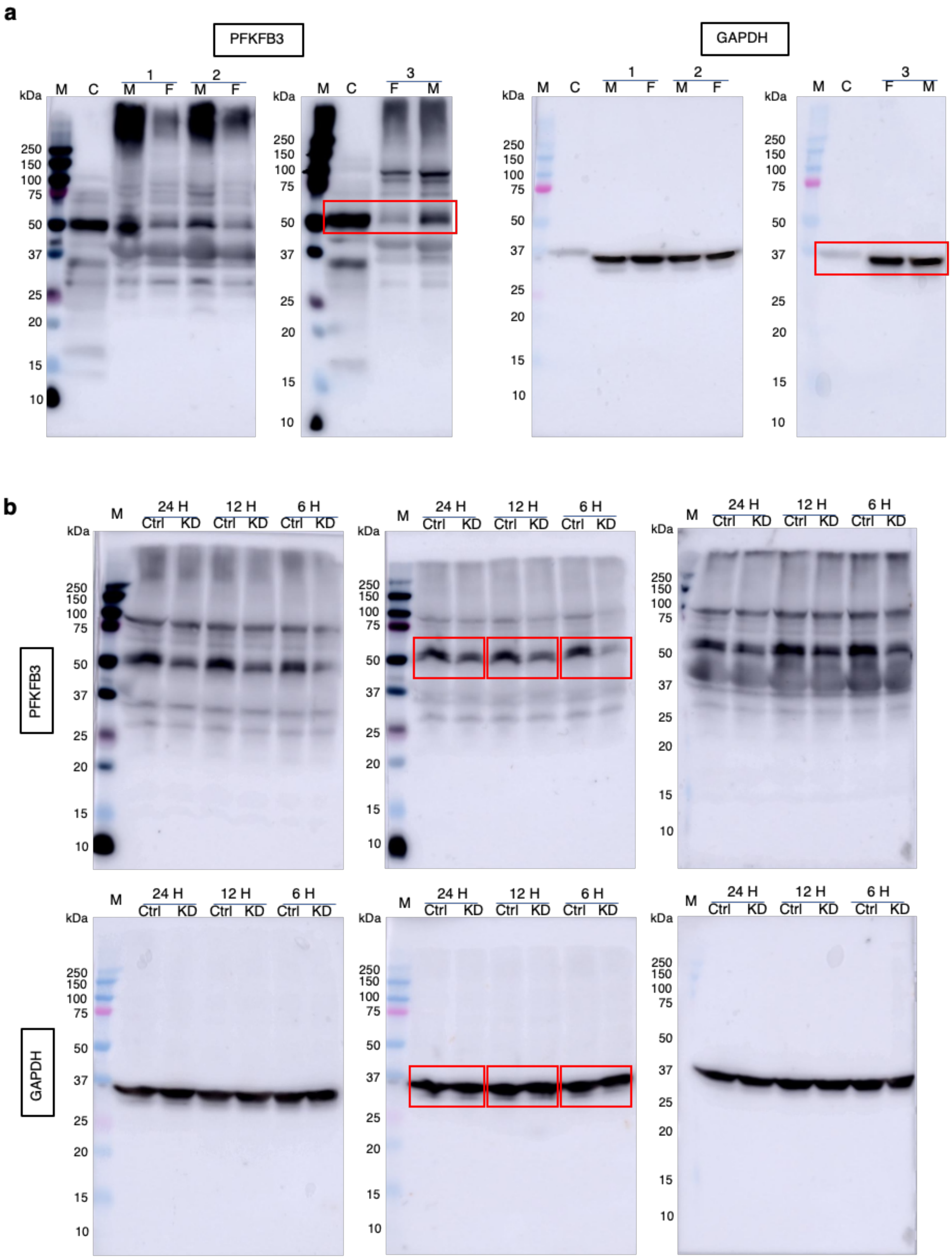
a
PFKFB3
GAPDH
kDa
M C 1 M F 2 M F
M C 3 F M
250 150 100 75 50 37 25 20 15 10
b
PFKFB3
GAPDH
M
24 H 12 H 6 H
Ctrl KD Ctrl KD Ctrl KD

c

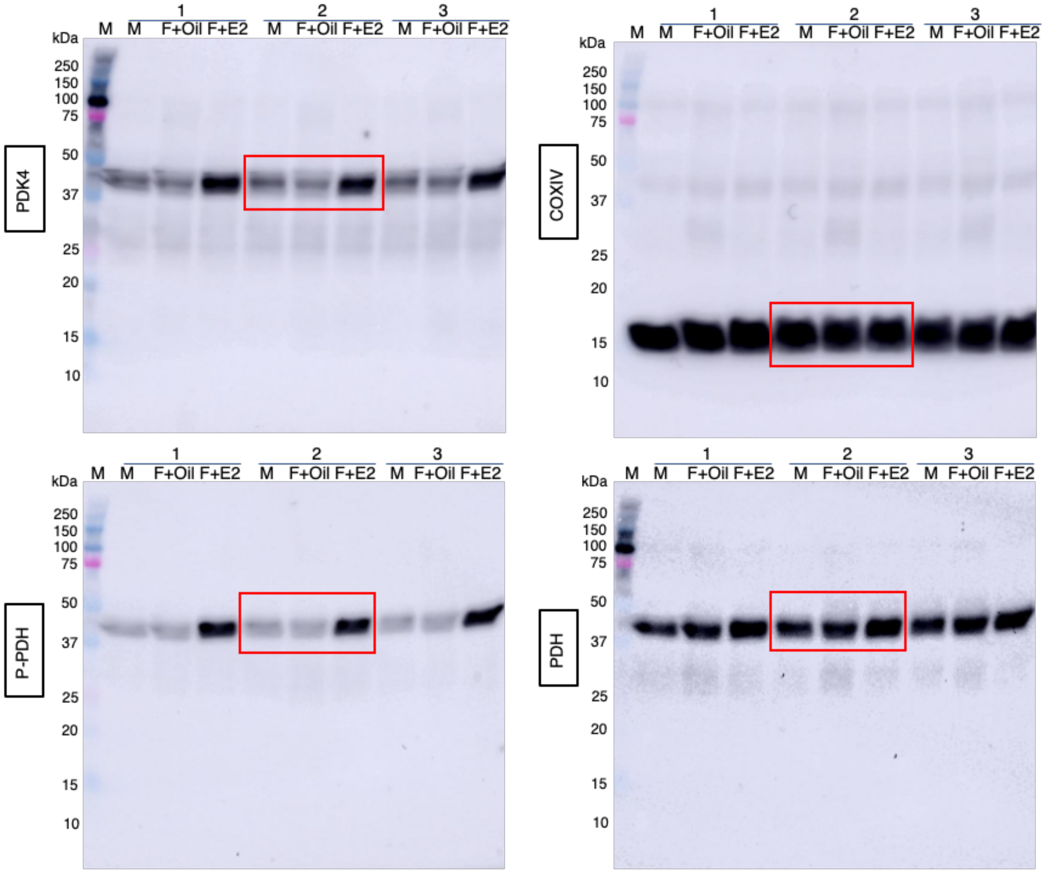
kDa
M
1
2
3
M F+Oil F+E2
PDK4
COXIV
P-PDH
PDH
250
150
100
75
50
37
25
20
15
10

d

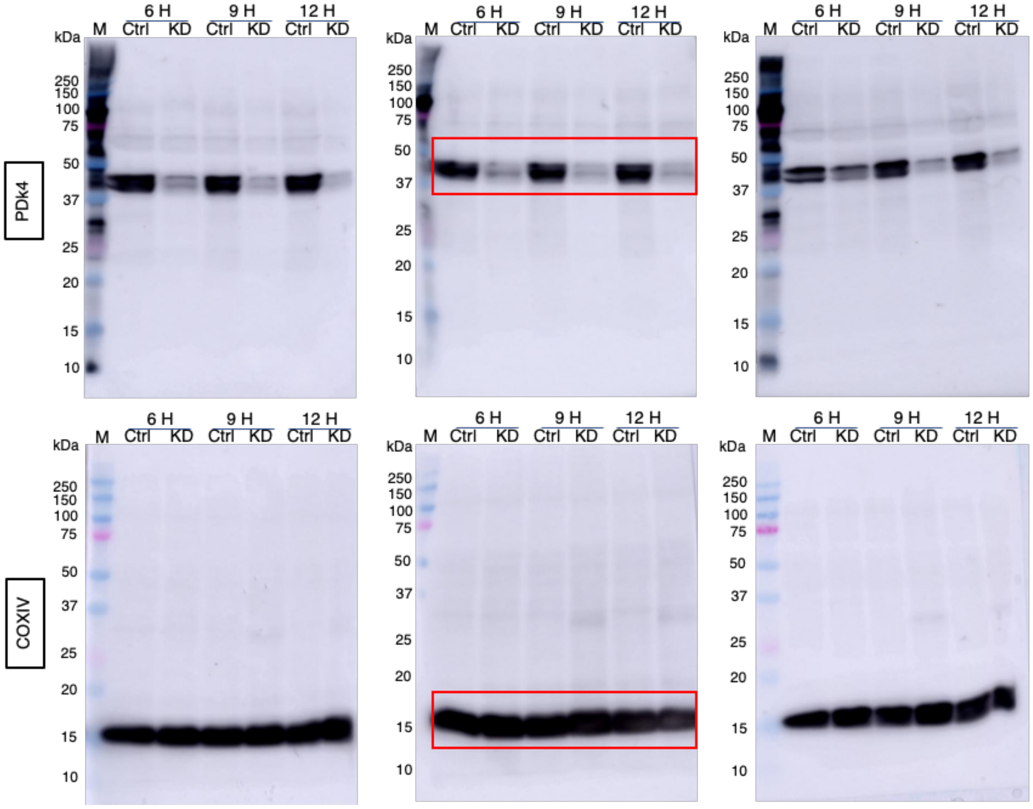
kDa
M
6 H
9 H
12 H
Ctrl KD
PDk4
COXIV
250
150
100
75
50
37
25
20
15
10

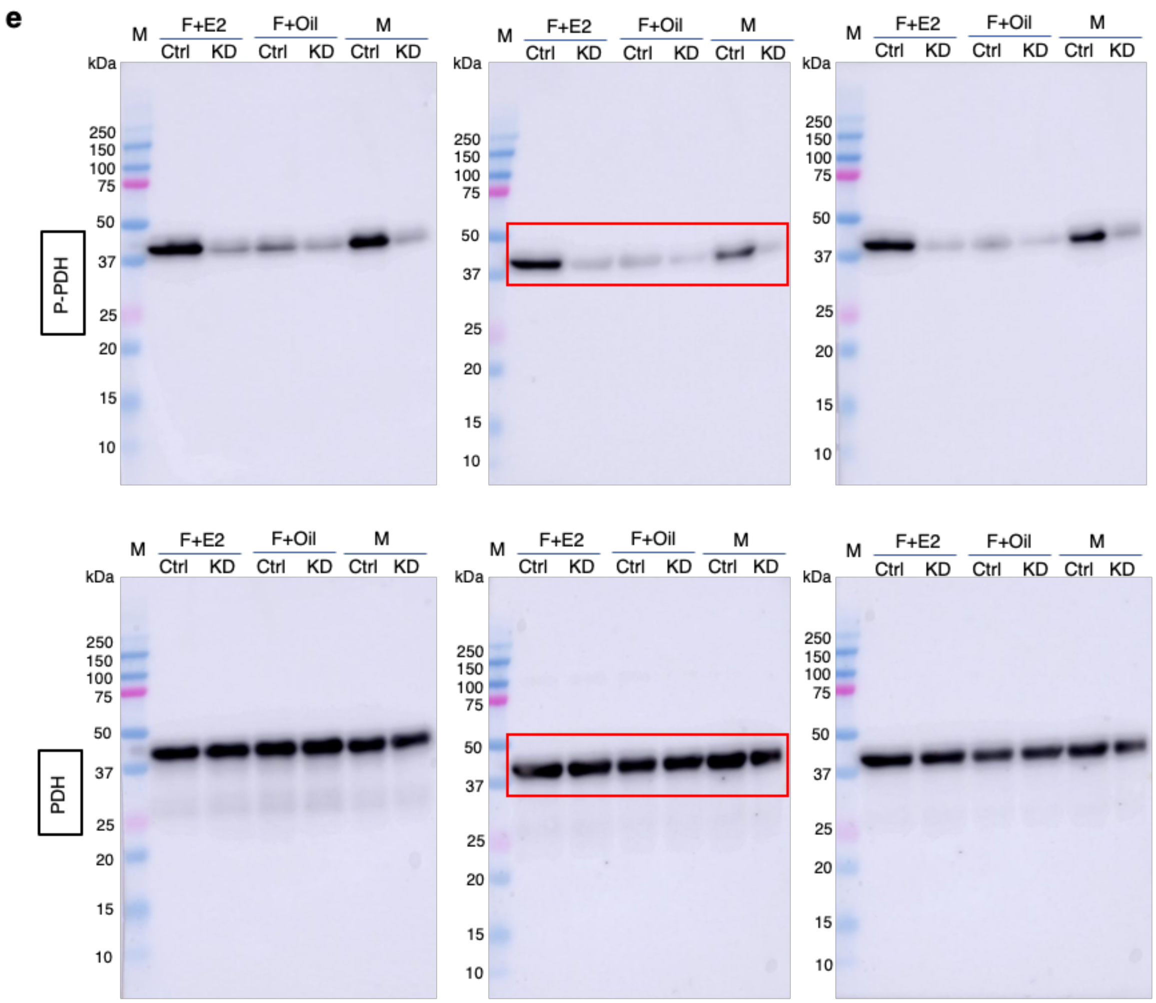

Supplementary Figure 5. Full blot images for cropped gels.

a) The boxed regions are used in Fig. 4f

b) The boxed regions are used in Fig. 5b

c) The boxed regions are used in Fig. 6d

d) The boxed regions are used in Fig. 6g

e) The boxed regions are used in Fig. 6h

Supplementary Table 1. List of GO terms related to male-enriched (upper) and female-enriched genes (lower) with $p < 10^{3}$.

Male-enriched genes

GO terms	p-value
Polyamine biosynthetic process	4.8E-04
Cholesterol homeostasis	1.2E-03
Positive regulation of activated T cell Proliferation	2.9E-03
Regulation of gene expression	3.5E-03
Response to hypoxia	3.8E-03
S-Adenosylmethioninamine biosynthetic process	6.6E-03
Response to oxidative stress	9.8E-03

Female-enriched genes

GO terms	p-value
Collagen fibril organization	1.8E-04
Wound healing	2.4E-03
Skeletal system development	3.4E-03
Positive regulation of catalytic activity	5.4E-03
Regulation of phosphorus metabolic process	8.4E-03

Supplementary Table 2. Male- and female-biased gene expression. a) The expressions of the polyamine synthetic genes in the 10 experimental groups are shown. The expressions of *Odc1*, *Amd1/2*, and *Smox* are higher in males than in females, and are induced by DHT treatment in both sexes. **b)** *Col*-family genes whose CPM values are higher than 10.0 in both males and females are shown. The expressions of *Col1a1*, *Col1a2*, *and Col3a1* are higher in females than in males by more than two-fold, and those of *Col5a2*, *Col5a3*, *Col6a2*, and *Col6a6* are higher in females than in males by more than 1.5-fold. The expressions of many *Col* genes are not largely affected by ovariectomy or sex steroid treatment in females, while they are induced by castration in males (compare Male vs Cas+P or Cas+O). **c)** The expressions of β-oxidation genes in the 10 experimental groups are shown. Several genes are induced by E2 treatment while the others are unaffected.

a

Gene	Male	Cas+P	Cas+DHT	Cas+O	Cas+E2	Female	Ovx+P	Ovx+DHT	Ovx+O	Ovx+E2
Odc1	137.3	46.1	155.7	43.1	36.1	47.0	46.7	103.3	35.7	35.2
Amd1/2	1561.7	153.5	1923.6	191.1	179.9	200.2	203.7	1596.0	169.5	146.4
Srm	4.8	8.5	4.3	6.0	6.4	3.4	5.0	5.6	4.5	4.2
Sms	110.1	79.0	99.1	84.2	80.6	110.7	85.6	93.1	85.2	75.9
Smox	468.6	58.7	363.0	117.7	90.6	106.5	105.1	533.6	123.6	80.1

b

Gene	Male	Cas+P	Cas+DHT	Cas+O	Cas+E2	Female	Ovx+P	Ovx+DHT	Ovx+O	Ovx+E2
Col1a1	95.1	193.7	127.3	184.9	244.1	248.5	258.3	253.9	222.6	267.1
Col1a2	90.4	158.4	175.1	156.5	182.0	191.0	230.3	281.8	187.5	231.8
Col3a1	159.7	392.3	395.0	337.6	227.0	386.1	482.5	733.4	413.1	307.0
Col4a1	158.2	303.5	323.7	239.4	176.2	197.6	273.5	364.8	235.8	268.0
Col4a2	110.0	205.3	187.6	156.2	112.0	118.8	190.3	224.1	159.5	180.1
Col5a1	44.1	69.1	73.5	57.8	49.6	65.5	71.9	120.3	65.4	65.6
Col5a2	19.7	38.8	39.5	29.0	25.4	32.0	38.4	61.6	34.9	36.5
Col5a3	43.8	61.3	81.1	49.3	45.7	71.2	85.3	121.1	79.4	88.6
Col6a1	34.4	73.4	67.5	46.7	45.2	39.0	88.9	90.6	69.5	77.2
Col6a2	30.8	74.9	71.0	60.0	52.1	47.2	89.4	101.0	71.5	87.3
Col6a3	77.1	161.5	131.4	120.3	95.6	107.9	190.4	183.0	140.3	169.2
Col6a6	14.1	20.6	24.4	18.3	4.0	13.3	26.1	30.4	22.9	9.9
Col7a1	21.0	25.3	55.4	22.4	18.1	18.0	21.2	49.1	19.5	13.0
Col15a1	39.3	117.7	86.1	77.8	33.0	53.2	100.0	72.1	86.4	81.1
Col22a1	13.8	51.7	27.7	21.0	56.3	17.7	30.6	17.7	17.8	41.6

c

Gene	Male	Cas+P	Cas+DHT	Cas+O	Cas+E2	Female	Ovx+P	Ovx+DHT	Ovx+O	Ovx+E2
Acsf3	13.63	11.95	11.69	15.92	12.54	13.03	11.73	13.77	12.42	13.69
Acsl1	158.20	120.46	148.18	103.13	147.88	137.67	122.59	120.09	127.54	221.43
Acsl3	59.53	33.31	58.20	22.86	26.68	26.15	19.80	39.08	17.47	15.89
Acsl4	37.49	36.56	41.58	40.47	33.98	41.10	33.86	33.00	37.04	37.93
Acsl6	44.98	44.53	47.20	45.89	42.84	41.05	41.74	45.96	42.34	34.82
Acss1	8.13	20.55	15.68	15.42	12.15	10.21	12.25	7.44	10.56	14.25
Acss2	15.41	19.58	17.72	16.47	16.96	15.25	11.80	13.40	17.07	24.63
Cpt1b	194.19	187.61	203.58	176.81	196.93	161.75	175.75	188.82	172.95	217.06
Cpt2	29.11	30.69	35.08	30.25	28.35	26.65	23.87	29.29	27.93	32.90
Slc25a20	20.65	22.62	20.57	14.03	22.15	15.35	16.25	16.56	18.93	30.81
Acad11	50.65	62.43	50.48	55.25	63.60	58.42	50.57	37.52	56.81	52.41
Acad12	14.59	21.78	17.32	17.80	19.23	14.31	15.91	14.53	18.37	13.05
Acad8	17.12	24.19	23.99	19.60	23.09	22.01	21.19	22.47	16.40	24.52
Acad9	24.15	21.95	25.50	25.21	24.79	22.75	22.55	25.14	23.80	27.08
Acadl	54.43	66.24	61.18	47.63	73.54	46.73	39.97	48.30	56.40	105.59
Acadm	104.55	95.78	115.06	83.48	110.30	78.31	75.75	84.38	86.88	123.38
Acads	27.64	36.18	35.24	36.23	42.82	32.71	30.09	38.49	34.46	37.24
Acadsb	77.73	79.91	75.07	85.14	89.96	82.31	88.54	59.26	83.95	94.77
Acadvl	100.48	116.30	119.52	105.62	134.64	100.22	104.79	106.64	110.91	160.56
Ech1	47.30	49.18	58.29	52.84	62.89	49.29	54.75	48.58	53.49	73.22
Echdc1	24.11	15.97	20.07	18.74	18.69	19.24	17.53	15.25	15.20	19.26
Echs1	31.50	60.47	38.27	50.07	54.04	34.44	41.93	29.17	41.22	52.55
Hadh	61.92	44.57	60.38	51.95	63.36	47.52	46.68	52.78	53.29	58.78
Hadha	165.01	148.77	153.96	145.95	165.29	139.94	136.99	151.93	146.50	185.27
Hadhb	135.55	140.63	147.37	133.60	143.49	126.47	118.78	125.38	123.08	149.82
Acaa1a	21.90	25.38	28.59	28.15	34.11	30.20	27.87	25.95	28.31	26.25
Acaa2	31.85	46.63	37.23	33.38	49.47	31.38	27.60	26.85	34.39	57.92

Supplementary Table 3. Nucleotide sequences of the primers used for RT-PCR

Gene	Forward (5' → 3')	Reverse (5' → 3')
Myh1	CCAAGTGCAGGAAAGTGACC	AGGAAGAGACTGACGAGCTC
Myh2	AAGCGAAGAGTAAGGCTGTC	GTGATTGCTTGCAAAGGAAC
Myh4	CAATCAGGAACCTTCGGAACAC	GTCCTGGCCTCTGAGAGCAT
Myh7	CCAAGGGCCTGAATGAGGAG	GCAAAGGCTCCAGGTCTGAG

Gene	Forward (5' → 3')	Reverse (5' → 3')
Actb	ATCCTGGCCTCACTGTCCACCTTC	AAACGCAGCTCAGTAACAGTCCGC
Aldoa	GCCGCAGCCAGTGAATCTCTCTTC	TTCACAGACAACACCGCACACGAG
Amd	GACGCATGAATTCTGACTGC	TGGGTCAAGCTCACTCATCA
Eno3	TCCCGTGGTCTCCATTGAG	CCACCCCAGAGAGGAATGAG
Gapdh	TGGTGGACCTCATGGCCTACATGG	TGAGGGAGATGCTCAGTGTTGGGG
Gpi1	AAGGAGGTGATGCAGATGCT	GCCCGATTCTCGGTGTAGT
Hk2	GCACTGGAGAAGAGCTTTTCGA	AGGGACACGCCCTTCATG
Pdk4	GGATTACTGACCGCCTCTTTAGTT	GCATTCCGTGAATTGTCCATC
Pfkfb3	AGAACTTCCACTCTCCCACCCAAA	AGGGTAGTGCCCATTGTTGAAGGA
Pfkm	CGATCTGTGGAGATGCTGAA	AATGGGATCAGATGCAAAGC
Pgam2	TACACCTCCATCAGCAAGGA	GCAATGGTGTCCTTGAGACTT
Pgk1	AAGTCCTTCCTGGGGTGGATGCTC	AGGGTTCCTGGTGCCACATCTCAG
Pkm	TTAGGCCAGCAACGCTTGTAGTGC	AGATGCTGCCGCCCTTCTGTGATA
Smox	GCCAGAGTGGAGAGAATCCG	CTGCCTCTTGAGCACGCCCA
Tpi1	TGAGCCGTTTCCACCGCCCTATTA	GCTCCAACCATGAGTTTCCAGCCC

Gene	Forward (5' → 3')	Reverse (5' → 3')
Acaa1	CCCTGCTACGAGGTGTGTTC	ACATTGCCCACGATGACACT
Acsd11	TGGCTAACATGTACGCCATCA	ATCTTGGCGATCGCTGAGA
Acadm	AGAGCTCTAGACGAAGCCAC	GAGTTCAACCTTCATCGCCATT
Acadsb	GTTGCTCCTCTGGTTTCCTCTAT	CCTCCATATTGTGCTTCAACTTC
Acadvl	GGAGGACGACACTTTGCAGG	AGCGAGCATACTGGGTATTAGA
Cpt1b	GTCGCTTCTTCAAGGTCTGG	AAGAAAGCAGCACGTTCGAT
Cpt2	GGATAAACAGAATAAGCACACCA	GAAGGAACAAAGCGGATGAG
Ech1	GCTACCGCGATGACAGTTTC	GCTCAGAGATCGAAGGCTGATG
Slc25a20	CATGTGCCTGGTGTTTGTGG	CCCTGTGATGCCCTCTCTCA
