# Supplementary material for: Sex differences in metabolic pathways are regulated by Pfkfb3 and Pdk4 expression in rodent muscle
Source: Commun Biol. 2021 Nov 4;4:1264. doi: 10.1038/s42003-021-02790-y (PMC8569015; doi:10.1038/s42003-021-02790-y)
Supplement: Supplementary file 3 — Description of Additional Supplementary Files [file 42003_2021_2790_MOESM3_ESM.pdf]

Description of Additional Supplementary Files

File name: Supplementary Data 1.
Description: Source data for graphs in Figure 1.

File name: Supplementary Data 2.
Description: Source data for graphs in Figure 3.

File name: Supplementary Data 3.
Description: Source data for graphs in Figure 4.

File name: Supplementary Data 4.
Description: Source data for graphs in Figure 5.

File name: Supplementary Data 5.
Description: Source data for graphs in Figure 6.
