# Supplementary material for: Sex differences in metabolic pathways are regulated by Pfkfb3 and Pdk4 expression in rodent muscle
Source: Commun Biol. 2021 Nov 4;4:1264. doi: 10.1038/s42003-021-02790-y (PMC8569015; doi:10.1038/s42003-021-02790-y)
Supplement: Supplementary file 9 — Reporting Summary [file 42003_2021_2790_MOESM9_ESM.pdf]

nature research

Corresponding author(s): Takashi Baba

Last updated by author(s): Sep 27, 2021

Reporting Summary

Nature Research wishes to improve the reproducibility of the work that we publish. This form provides structure for consistency and transparency in reporting. For further information on Nature Research policies, see our Editorial Policies and the Editorial Policy Checklist.

Statistics

For all statistical analyses, confirm that the following items are present in the figure legend, table legend, main text, or Methods section.

n/a	Confirmed	
☐	☒	The exact sample size (*n*) for each experimental group/condition, given as a discrete number and unit of measurement
☐	☒	A statement on whether measurements were taken from distinct samples or whether the same sample was measured repeatedly
☐	☒	The statistical test(s) used AND whether they are one- or two-sided<br>*Only common tests should be described solely by name; describe more complex techniques in the Methods section.*
☒	☐	A description of all covariates tested
☐	☒	A description of any assumptions or corrections, such as tests of normality and adjustment for multiple comparisons
☐	☒	A full description of the statistical parameters including central tendency (e.g. means) or other basic estimates (e.g. regression coefficient) AND variation (e.g. standard deviation) or associated estimates of uncertainty (e.g. confidence intervals)
☐	☒	For null hypothesis testing, the test statistic (e.g. *F*, *t*, *r*) with confidence intervals, effect sizes, degrees of freedom and *P* value noted<br>*Give P values as exact values whenever suitable.*
☒	☐	For Bayesian analysis, information on the choice of priors and Markov chain Monte Carlo settings
☒	☐	For hierarchical and complex designs, identification of the appropriate level for tests and full reporting of outcomes
☒	☐	Estimates of effect sizes (e.g. Cohen's *d*, Pearson's *r*), indicating how they were calculated

Our web collection on statistics for biologists contains articles on many of the points above.

Software and code

Policy information about availability of computer code

Data collection	ImageJ software (Fiji) was used to collect data for cross-sectional area (CSA) from muscle histology and for protein quantification from western blotting results. STAR (version 2.7.3a) and featureCounts (version 2.0.0) were used for alignment and assembly of the sequence read, respectively.
Data analysis	Microsoft Excel (Version 16.41) was used to generate box-and-whisker plots and bar graphs and to analyze data statistically. Basical Fiji functions were used for calculation of CSAs and for quantification of western blotting band intensities. RStudio (Version 1.1.463) with R (Version 3.6.0) was used for analyzing sequencing data, making PCA plots, and drawing heat-maps.

For manuscripts utilizing custom algorithms or software that are central to the research but not yet described in published literature, software must be made available to editors and reviewers. We strongly encourage code deposition in a community repository (e.g. GitHub). See the Nature Research guidelines for submitting code & software for further information.

Data

Policy information about availability of data

All manuscripts must include a data availability statement. This statement should provide the following information, where applicable:

- Accession codes, unique identifiers, or web links for publicly available datasets
- A list of figures that have associated raw data
- A description of any restrictions on data availability

Raw data that is not already presented in the figures or supplementary figures are available upon reasonable request to the corresponding author. Correspondence and request for those materials should be addressed to T.B.

Field-specific reporting

Please select the one below that is the best fit for your research. If you are not sure, read the appropriate sections before making your selection.

☒ Life sciences ☐ Behavioural & social sciences ☐ Ecological, evolutionary & environmental sciences

For a reference copy of the document with all sections, see nature.com/documents/nr-reporting-summary-flat.pdf

Life sciences study design

All studies must disclose on these points even when the disclosure is negative.

Sample size	Sample sizes for each experiment are included in the legend or explained in the material and methods section.
Data exclusions	For CSA analysis, positive MYH2B expression with the area below 100 µm square and above 10,000 µm square were excluded from the calculation.
Replication	All data shown were replicated independently with the representative of N=3 replicates. Details are explained in the methods section.
Randomization	Mice were randomly assigned to the 10 experimental mouse groups (sham-operated males and females, males and females transplanted with a DHT-containing or empty pellet after gonadectomy, and males and females injected with E2-containing or corn oil after gonadectomy). In addition, for OCR, ECAR, knockdown experiments, and western blotting analysis, mice were randomly assigned to male mice, oil-injected female mice in diestrus, and female mice treated with E2 for 24 h before isolating the myofibers and subjected for culture procedure. Detailed randomization groups are explained in the manuscript and supplementary figure 2.
Blinding	In all experiments, the investigator was not blinded to the treatment. Furthermore, all the analyses of this experiment are done by software or computer algorithms and should not be subjected to investigator bias.

Reporting for specific materials, systems and methods

We require information from authors about some types of materials, experimental systems and methods used in many studies. Here, indicate whether each material, system or method listed is relevant to your study. If you are not sure if a list item applies to your research, read the appropriate section before selecting a response.

Materials & experimental systems

n/a	Involved in the study	
☐	☒	Antibodies
☒	☐	Eukaryotic cell lines
☒	☐	Palaeontology and archaeology
☐	☒	Animals and other organisms
☒	☐	Human research participants
☒	☐	Clinical data
☒	☐	Dual use research of concern

Methods

n/a	Involved in the study	
☒	☐	ChIP-seq
☒	☐	Flow cytometry
☒	☐	MRI-based neuroimaging

Antibodies

Antibodies used	The List of all antibodies used in this experiment is explained in the methods section. For immunofluorescence analysis, antibodies against MYH2B (myosin heavy chain type IIB) (1:1000), MYH2A (myosin heavy chain type IIA) (1:1000), and laminin (1:1000) (Sigma-Aldrich, St. Louis, MO, USA) were used as the primary antibodies. Mouse Anti-Rat IgG2b-Alexa Fluor® 647 (1:500, SouthernBiotech, Birmingham, AL, USA), Mouse Anti-Rat IgG1-Alexa Fluor 488® (1:500, SouthernBiotech), and Alexa Fluor 488-labeled Goat Anti-Rabbit IgG (1:500, Thermo Fisher Scientific, Waltham, MA, USA) were used as the secondary antibodies. And for western blotting analysis, anti-PFKFB3 (1:2000, Proteintech, Rosemont, IL, USA), anti-GAPDH (1:10000, Santa Cruz Biotechnology, Dallas, Texas, USA), anti-PDK4 (1:1000, Proteintech), anti-PDH (1:1000, Cell Signaling Technology, Danvers, MA, USA), anti-phospho-PDH α1 (1:1000, Cell Signaling Technology), and anti-COXIV antibodies (1:2000, Abcam, Cambridge) were used as primary antibodies. HRP labeled anti-mouse IgG (Goat) (1:2000, Thermo Fisher Scientific) and HRP-linked F(ab')2 fragment of anti-Rabbit IgG (Donkey) (1:2000. Cytiva, Marlborough, MA, USA) were used as secondary antibodies.
Validation	The vendor validated all antibodies for immunofluorescence and western blotting analysis. Furthermore, the antibodies used for immunofluorescence were validated extensively in the publication cited in the Methods section (Sawano et al., 2019).

Animals and other organisms

Policy information about studies involving animals; ARRIVE guidelines recommended for reporting animal research

Laboratory animals	Male and female C57BL/6J mice were bought from Japan SLC Inc. at 3 weeks old. Treatment with DHT and E2 was performed as explained in the methods section.
Wild animals	This study did not involve wild animals.
Field-collected samples	This study did not involve samples collected from the field.
Ethics oversight	All mouse experiment protocols were approved by the Animal Care and Use Committee of Kyushu University (Fukuoka, Japan).

Note that full information on the approval of the study protocol must also be provided in the manuscript.
